# A meta-analysis of obesity and the risk of pancreatic cancer

**DOI:** 10.1038/sj.bjc.6601140

**Published:** 2003-07-29

**Authors:** A Berrington de Gonzalez, S Sweetland, E Spencer

**Affiliations:** 1 Cancer Research UK Epidemiology Unit, University of Oxford, Gibson Building, Radcliffe Infirmary, Oxford OX2 6HE, UK

**Keywords:** pancreatic neoplasms, risk factors, obesity, body mass index, anthropometry

## Abstract

Smoking and diabetes are the only established risk factors for pancreatic cancer. Findings from recent studies suggest that obesity may also be associated with an increased risk of pancreatic cancer, but several earlier studies were less conclusive. We examined this relationship in a meta-analysis of published data. Six case–control and eight cohort studies involving 6391 cases of pancreatic cancer were identified from a computer-based literature search from 1966 to 2003. The relative risk per unit increase in body mass index was estimated for each of the studies from the published data. In a random effects model, the summary relative risk per unit increase in body mass index was 1.02 (95% CI: 1.01–1.03). There was some evidence of heterogeneity between the studies' results (*P*=0.1). The summary relative risk estimates were slightly higher for studies that had adjusted for smoking and for case–control studies that had not used proxy respondents. The estimated per unit increase in body mass index would translate into a relative risk of 1.19 (95% CI: 1.10–1.29) for obese people (30 kg m^−2^) compared to people with a normal body weight (22 kg m^−2^). These results provide evidence that the risk of pancreatic cancer may be weakly associated with obesity. However, the small magnitude of the summary risk means the possibility of confounding cannot be excluded.

Pancreatic cancer is the fifth most common cause of cancer mortality in the European Union and North America and is responsible for approximately 70 000 deaths in these regions annually (Ferlay *et al*, 2001). Smoking and diabetes are the only well-established risk factors for this invariably fatal disease (Gapstur and Gann, 2001). Smoking, however, is thought to account for at most 20–40% of pancreatic cancer cases (International Agency for Research on Cancer, 1990). The findings from several large US cohort studies suggest that obesity (defined as body mass index of at least 30 kg m^−2^) may also be a risk factor for pancreatic cancer (Michaud *et al*, 2001; Calle *et al*, 2003). The results of several earlier observational studies were less conclusive. The aim of this meta-analysis was to examine the relationship between pancreatic cancer and body mass index in relevant published epidemiological studies.

## MATERIALS AND METHODS

### Data sources

Epidemiological studies were identified through searches of the electronic databases MEDLINE (1966–2003), EMBASE (1980–2003) and the Science Citation Index (1981–2003) and also from citations in the selected papers and review articles. The key words that were used for the search were pancreatic cancer, obesity, body mass index, anthropometric factors and risk factors. The search was limited to human studies and restricted to peer-reviewed articles. No language or date limitations were imposed.

### Study selection

Each study was required to have published information on the number of study participants and on age-adjusted or age-matched relative risks (or odds ratios, subsequently referred to as relative risks), and their corresponding confidence intervals according to categories of body mass index or per unit increase in body mass index, or data that would allow this to be estimated. Details of the boundaries that were used for the categories of body mass index were also required.

In total, 16 studies were identified in the search of which 14 satisfied the criteria for inclusion. A US case–control study (Lyon *et al*, 1993) was not included because the authors only published a crude relative risk for the top tertile to the lowest tertile of body mass index, and did not provide any information on the boundaries for the tertiles. A Chinese case–control study (Ji *et al*, 1996) was excluded because there is evidence that adiposity-associated health effects occur at lower levels of body mass index among Asian populations than among Western populations (WHO, IASO and IOT, 2000). The studies that were included were all of either European or North American populations.

### Data extraction

For each eligible study, the following information was extracted independently by two reviewers: country and year of diagnosis of the cases; study design (cohort or case–control and type of controls); measured or self-reported weight; at what time point body weight was assessed; whether proxy respondents had been used; categories of body mass index; relative risk and 95% confidence intervals for each category of body mass index; estimated relative risk per unit increase in body mass index; adjustment factors used in the analysis. The most fully adjusted relative risks were extracted from each published article.

### Statistical methods

Body mass index was defined as weight height^−2^ in all the studies except one. Silverman *et al* calculated body mass index as weight height^−1.5^ for women only. To translate these categories of body mass index into weight height^−2^, we divided the body mass index categories by the square root of height where height was set to 1.64 m, the mean height of women in a large US cohort study (Michaud *et al*, 2001).

Only three studies (Friedman and van den Eeden, 1993; Michaud *et al*, 2001) had reported results in the form of a relative risk per unit increase in body mass index. Therefore, for the other studies it was necessary to estimate the relative risk per unit increase in body mass index by regressing the risks reported according to categories of body mass index on the mid-point of each category. This was done using weighted least squares regression analysis with the weights taken as the variance of the log relative risk. Adjustment was made for the lack of independence of risks within each study (because the risks are all estimated with the same baseline) using a method for combining nonindependent strata (Berrington and Cox, 2003). For any open-ended categories of body mass index (e.g. <25 kg m^−2^), the mid-points were estimated assuming that body mass index was normally distributed and by taking the mid-point between the specified boundary and the estimated first percentile for estimation of a lower limit or 99th percentile for an upper limit. A sensitivity analysis was conducted to investigate the effect of the choice of boundaries on the slope estimates. Two case–control studies (Howe *et al*, 1990; Zatonski *et al*, 1991) only published mean values for body mass index for cases and controls. Approximate slope estimates were calculated for these studies using the method described by Greenland (1987).

Relative risks for each study are plotted as black squares whose size is inversely proportional to the variance of the logarithm of the relative risk. Diamonds represent summary relative risks for the pooled data, calculated using the method of empirically weighted least squares where the weights are defined as the inverse of the variance of the log relative risks under a random effects model (Cox, 1977). All but five of the studies had reported results for male and female patients separately. The results for Howe *et al* were also reported for proxy and nonproxy respondents separately. To examine other potential sources of variability, summary results were stratified according to sex and other study design and adjustment factors. Heterogeneity between these factors was estimated using a meta-regression model (Ursin *et al*, 1995).

## RESULTS

Overall six case–control (Bueno de Mesquita *et al*, 1990; Howe *et al*, 1990; Ghadirian *et al*, 1991; Zatonski *et al*, 1991; Silverman *et al*, 1998; Hanley *et al*, 2001) and eight cohort studies (Friedman and van den Eeden, 1993; Shibata *et al*, 1994; Gapstur *et al*, 2000; Michaud *et al*, 2001; Stolzenberg-Solomon *et al*, 2002; Calle *et al*, 2003; Lee *et al*, 2003) were eligible for inclusion in this meta-analysis. In total, these studies included 6391 cases of pancreatic cancer (Table 1

**Table 1:** Details of the 14 studies included in the meta-analysis

**Study first author *Year* (Country)**	**Study design**	**Period of diagnosis**	**Time of weight estimate**		**Number of**			**Adjustments**	
				**Sex**	**Cases**	**Controls**	**Proxies**	**Smoking**	**Diabetes**
Bueno de Mesquita 1990 (Netherlands)	C–C	1984–1988	2 years before interview	M	90	232	Yes	Yes	No
				F	74	248			
Howe 1990 (Canada)	C–C	1983–1986	2 years before interview	M	141	270	Yes	No	No
				F	108	235			
Ghadirian 1991 (Canada)	C–C	1984–1988	2 years before interview	M, F	179	239	Yes	Yes	No
Zatonski 1991 (Poland)	C–C	1985–1988	Not known	M, F	110	195	Yes	No	No
Friedman 1993 (USA)	Cohort	1945–1988	Prospective (measured)	M, F	450	2687	NA	Yes	No
Shibata 1994 (USA)	Cohort	1981–1990	Prospective	M, F	65	NA	NA	Yes	No
Silverman 1998 (USA)	C–C	1986–1989	Usual in adulthood	M	218	1231	No	Yes	Yes
				F	213	747			
Gapstur 2000 (USA)	Cohort	1967–1995	Prospective (measured)	M	96	NA	NA	Yes	Yes
				F	43	NA			
Michaud 2001 (USA)	Cohort	1976–1998	Prospective	M	140	NA	NA	Yes	Yes
				F	210	NA			
Hanley 2001 (Canada)	C–C	1994–1997	2 years before interview	M	173	1074	No	Yes	No
				F	139	1191			
Stolzenberg-Solomon 2002 (Finland)	Cohort	1985–1997	Prospective (measured)	M	172	NA	NA	NA	Yes
Lee 2003 (USA)	Cohort	1962–1995	Prospective	M, F	212	NA	NA	NA	Yes
Calle 2003 (USA)	Cohort	1982–1998	Prospective	M	1908	NA	NA	Yes	No
				F	1650	NA			

NA=not applicable; C–C=case–control study; M=males; F=females.

Michaud (2001) published the results of two cohort studies in one paper. The male subjects were one cohort study (Health Professionals' Follow-up study) and the female subjects were the other (Nurses' Health Study).

). Most of the studies (11) were of North American populations. Height and weight were measured in three of the cohort studies and were self-reported in all the other studies. All of the case–control studies used population-based controls. Four of the case–control studies used proxy respondents because some of the cases were too ill to be interviewed.

**Figure 1:**
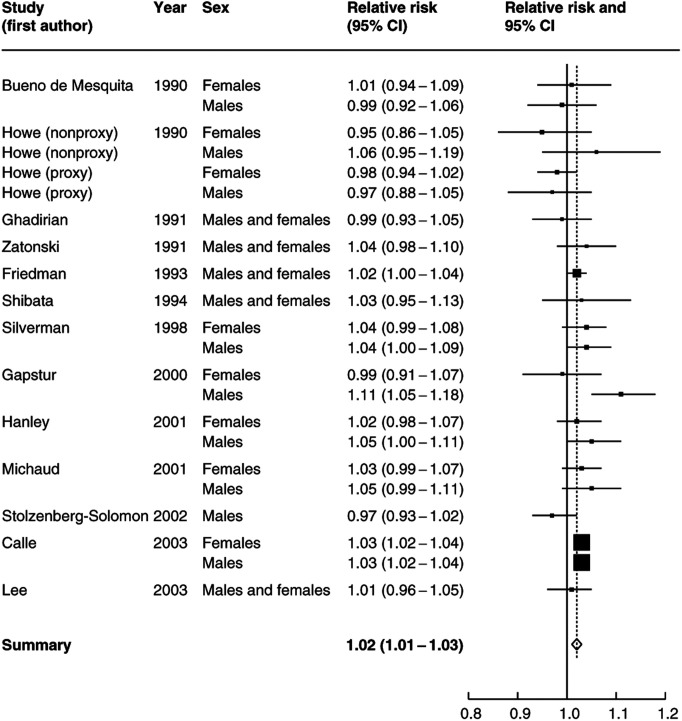
Estimated relative risk and 95% confidence interval (95% CI) of pancreatic cancer for a unit increase in body mass index.

The estimated increase in relative risk per unit increase in body mass index for each of the 14 studies is shown in Figure 1

separately for male and female subjects (wherever this information was available). There was some evidence of heterogeneity between the estimates (*P*=0.1), with 15 of the 22 estimates suggesting a positive association between body mass index and the risk of pancreatic cancer and the remainder a negative one. Overall, there was evidence of a small positive increase in risk per unit increase in body mass index and the summary relative risk was 1.02 (95% CI: 1.01–1.03). This per unit increase would be equivalent to a relative risk of 1.19 (95% CI: 1.10–1.29) for obese people (30 kg m^−2^) compared to people with a normal body weight (22 kg m^−2^). The results that had the greatest influence on the summary risk were the female subjects from the study by Howe (using proxy respondents), and the studies by Stolzenberg-Solomon and Calle. In particular, if the female patients from the Howe (proxy respondents) study were excluded, then the summary relative risk increased to 1.03.

For most of the studies, the risk per unit increase in body mass index had to be estimated from published data. To assess the sensitivity of the summary relative risk to the estimation of the mid-points for the open-ended categories of body mass index (e.g. <25 kg m^−2^), the categories were reassigned mid-points that were 5, 10 and 15% below (for the bottom category or above for the top category) the specified boundary. For example, if the baseline category was <23 kg m^−2^, then the category mid-point was set at 21.85, 20.70 and 19.55 kg m^−2^ and the relative risk per unit increase in body mass index was recalculated. The overall summary relative risk was quite insensitive to these changes. The use of 5% above and below the bottom and top category increased the summary relative risk to 1.03 (95% CI: 1.01–1.04), whereas 15 and 10% left it unchanged).

**Figure 2:**
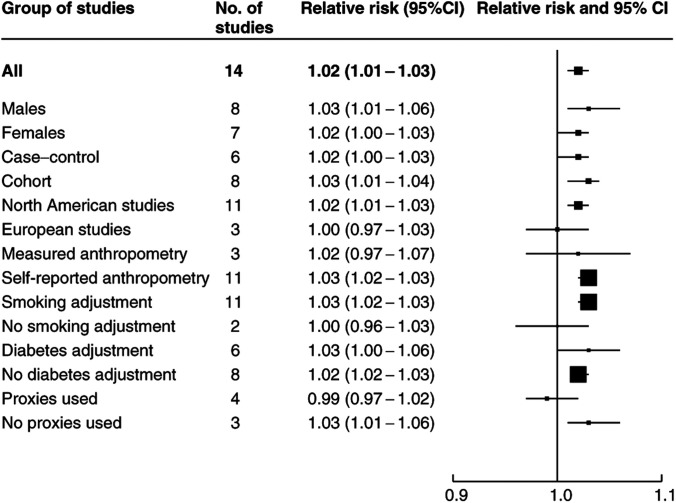
Estimated relative risk and 95% confidence interval (95% CI) of pancreatic cancer for a unit increase in body mass index in different groups of studies.

To explore the sources of heterogeneity between the studies, the relative risk estimates were calculated for subgroups of studies defined according to various covariates (see Figure 2

). The summary relative risk for a unit increase in body mass index was marginally higher for male than for female subjects (1.03 compared to 1.02) and also for cohort compared to case–control studies (1.03 compared to 1.02). The results from the North American studies were also higher than the European studies (1.02 and 1.00, respectively). Studies in which height and weight had been measured had a slightly lower summary relative risk (1.02) than studies in which they had been self-reported (1.03). When we restricted the studies to those that had adjusted for smoking, the summary relative risk increased to 1.03. Finally, although diabetes is now an established risk factor for pancreatic cancer some of the studies had not adjusted for diabetes. In the six studies that had made this adjustment, the summary per unit increase in relative risk was 1.03 compared to 1.02 in the studies that had not adjusted for diabetes. Tests for heterogeneity between each of these summary results generally lacked power; however, there was some evidence of significant differences in the summary relative risk according to whether an adjustment for smoking had been made (*P*=0.04). Some of the case–control studies had used proxy respondents if cases were too ill to be interviewed and there was evidence also of some heterogeneity between these risk estimates (*P*=0.02). In the studies that had used proxy respondents, the summary relative risk was lower (0.99) than in those that did not use proxy respondents (1.03).

## DISCUSSION

Overall, there was evidence of a small positive increase in the risk of pancreatic cancer per unit increase in body mass index. A unit increase in body mass index for a male patient of 1.78 m (5 ft 10 in) represents weight gain of about 3 kg (6.6 lbs). For a female patient of 1.64 m (5 feet 5 inches), a unit increase in body mass index represents weight gain of about 2.5 kg (5.5 lbs). The observed per unit increase in relative risk translates into a 19% higher risk of pancreatic cancer for obese people (body mass index >30 kg m^−2^) compared to those of normal body weight (22 kg m^−2^). There was, however, some evidence of heterogeneity between the studies' results (*P*=0.1). The summary relative risk estimates were slightly higher for studies that had adjusted for smoking and for case–control studies that had not used proxy respondents.

As this meta-analysis was based on published data, there is the possibility that publication bias could have affected the results. The relative risks are presented in chronological order of publication, and there was no visual evidence of publication bias (Figure 1). Neither was there evidence that the smaller studies tended to have positive results more frequently. However, it is not possible to rule out the possibility that further data, which show no evidence of an association between body mass index and pancreatic cancer risk, exist but have not been published.

Two case–control studies that had published information on body mass index and the risk of pancreatic cancer could not be included in this analysis. A US case–control study of 149 cases of pancreatic cancer conducted in Utah (Lyon *et al*, 1993), which was not included because the authors only published crude relative risks for the highest to lowest tertile and provided no information on the tertile boundaries, found a relative risk for the highest tertile of body mass index compared to the lowest of 0.83 (95% CI: 0.43–1.58) for men and 1.05 (95% CI: 0.53–2.08) for women. Although the study findings were null, the CI were wide and were therefore not inconsistent with our summary relative risk of 1.19 for obese people (body mass index >30 kg m^−2^) compared to those of normal body weight (22 kg m^−2^). A Chinese case–control study (Ji *et al*, 1996) was not included because there is evidence that adiposity-associated health effects occur at lower levels of body mass index among Asian populations than among Western population (WHO, IASO and IOT, 2000). It was not clear therefore that a relative risk per unit increase in body mass index would have the same meaning in an Asian as in a Western population. In this study, which included 483 cases of pancreatic cancer, there was evidence of an increasing risk of pancreatic cancer with increasing body mass index in men (*P*=0.05) but not in women (*P*=0.31). The relative risk for the top quartile of body mass index in male patients (>22.4 compared to <19.5 kg m^−2^) was 1.38 (95% CI: 0.91–2.08) and for female patients, it was 1.46 (95% CI: 0.85–2.51) (>23.1 kg m^−2^ compared to <19.5 kg m^−2^).

The studies that were included in this meta-analysis contained 91% of the cancer cases that were available and the results from the two studies that could not be included were not inconsistent with the findings from this meta-analysis. Two other record linkage cohort studies of obese individuals both found an elevated risk of pancreatic cancer compared to expected rates in the general population of 1.7 and 1.5 (95% CI: 1.1–1.9), respectively (Moller *et al*, 1994; Wolk *et al*, 2001). The study by Wolk *et al* found that the risk decreased with age from 2.5 (95% CI: 1.5–4.0) for those aged <60 years, to 2.0 (1.3–2.8) for 60–69 year olds and 0.7 (0.4–1.2) for those aged 70+years.

Smoking and diabetes are both potential confounding factors for the relationship between obesity and pancreatic cancer. If there is an association between body mass index and smoking, however, it is more likely to be a negative one, as current smokers have been observed to weigh less than nonsmokers (Istvan *et al*, 1992). Therefore, ignoring confounding by smoking could make the association between obesity and pancreatic cancer less positive. Only two of the studies that were included in this meta-analysis had not adjusted the risk estimates for smoking (Howe *et al*, 1990; Zatonski *et al*, 1991). When these studies and the cohort study of smokers (Stolzenberg-Solomon *et al*, 2002) were excluded, the summary relative risk per unit increase in body mass index was slightly higher (1.03). In the cohort study by Calle *et al*, the risk associated with obesity was investigated in men and women who had never smoked. In this subgroup, the relative risk of pancreatic cancer mortality for a body mass index of 35–39.9 kg m^−2^ was 2.61 compared to those with a normal body mass index (18.5–24.9 km m^−2^). In the analysis with adjustment for smoking, the relative risks were somewhat lower at 1.49 for men and 1.41 for women.

Long-standing diabetes has also been established as a risk factor for pancreatic cancer with duration of diabetes of 5 years or more being associated with a two-fold increased risk of pancreatic cancer (Everhart and Wright, 1995). Hence, a history of diabetes could positively confound the relationship between the risk of pancreatic cancer and body mass index. However, in the six studies in this meta-analysis that had adjusted for a history of diabetes, the risks associated with a unit increase in body mass index were actually marginally higher than in the studies that had not adjusted for this risk factor (1.03 compared to 1.02). In their case–control study, Silverman *et al* also published the risks associated with obesity cross-classified by a history of diabetes. Although there was evidence of an increase in risk for both those with and without the disease, within each level of body mass index diabetics had a higher risk of pancreatic cancer than nondiabetics (Silverman *et al*, 1999). Future studies need to examine the relationship between obesity and pancreatic cancer in more detail in those who have never smoked and in those without a history of diabetes.

All of the studies except for Gapstur *et al*, Friedman *et al* and Stolzenberg-Solomon *et al* relied upon self-reported height and weight and it is possible that weight may have been under-reported, especially by overweight or obese individuals (Spencer *et al*, 2002). Such under-reporting could result in overestimation of the dose–response relationship. The summary relative risk estimate for the studies that had measured anthropometry was marginally lower than those that relied upon self-reporting (1.02 *vs* 1.03). In case–control studies, under-reporting of weight could be a potential bias if it occurred unequally among cases and controls. Case–control studies could also be biased if the individuals in the control group were more ‘health conscious’ and thus less likely to be overweight than the cases. However, the summary relative risks in the case–control studies (1.02) were actually slightly lower than those for the cohort studies (1.03).

Obesity may be related to an increased risk of several other cancers including those of the endometrium, colorectum, oesophagus, kidney and postmenopausal breast cancer (IARC, 2002). Some of the mechanisms that have been suggested to explain these relationships may also be relevant for pancreatic cancer, including the hypothesis that insulin resistance and abnormal glucose metabolism may be a factor in pancreatic cancer development (Gapstur *et al*, 2000). The association between diabetes and pancreatic cancer risk (Everhart and Wright, 1995) supports this hypothesis, and further support for this is given by findings that physical activity may be associated with a decreased risk of pancreatic cancer (Hanley *et al*, 2001; Michaud *et al*, 2001).

This meta-analysis of the available observational data provides evidence that the risk of pancreatic cancer may increase slightly with increasing body mass index, and that obese individuals may have a risk that is 19% higher than those with a normal body mass index. However, the small magnitude of the summary relative risk means that the possibility of confounding cannot be excluded.
